# Interactions between two parasites of brown trout (*Salmo trutta*): Consequences of preinfection

**DOI:** 10.1002/ece3.4406

**Published:** 2018-09-29

**Authors:** Mikhail Gopko, M. Motiur R. Chowdhury, Jouni Taskinen

**Affiliations:** ^1^ A.N. Severtsov Institute of Ecology and Evolution Russian Academy of Sciences Moscow Russia; ^2^ Department of Biological and Environmental Science University of Jyväskylä Jyvaskyla Finland

**Keywords:** community ecology, *Diplostomum*, experimental infection, freshwater pearl mussel, host–parasite interactions, multiple infections

## Abstract

Preinfection by one parasitic species may facilitate or by contrast hamper the subsequent penetration and/or establishment of other parasites in a host. The biology of interacting species, timing of preinfection, and dosage of subsequent parasite exposure are likely important variables in this multiparasite dynamic infection process. The increased vulnerability to subsequent infection can be an important and often overlooked factor influencing parasite virulence. We investigated how the preinfection by freshwater pearl mussel *Margaritifera margaritifera* glochidia could influence the success of subsequent infection by the common trematode *Diplostomum pseudospathaceum* in brown trout *Salmo trutta* and *vice versa* whether preinfection by the trematode made fish more susceptible to glochidia infection. The first experiment was repeated twice with different (low and high) exposure doses to initiate the subsequent trematode infection, while in the second experiment we varied the timing of the preinfection with trematodes. The preinfection with glochidia made fish more vulnerable to subsequent infection with trematodes. Since the trematodes penetrate through the gills, we suggest that increased host vulnerability was most likely the result of increased respiration caused by the freshwater pearl mussel glochidia encysted on gills. In turn, brown trout preinfected with trematodes were more vulnerable to the subsequent glochidial infection, but only if they were preinfected shortly before the subsequent infection (20 hr). Fish preinfected with trematodes earlier (2 weeks before the subsequent infection) did not differ in their vulnerability to glochidia. These effects were observed at moderate intensities of infections similar to those that occur in nature. Our study demonstrates how the timing and sequence of exposure to parasitic species can influence infection success in a host–multiparasite system. It indicates that the negative influence of glochidia on host fitness is likely to be underestimated and that this should be taken into consideration when organizing freshwater pearl mussel restoration procedures.

## 1 INTRODUCTION

Individuals of free‐living organisms are usually infected by several parasite species. However, a majority of host–parasite studies are concentrated around single parasite and single host interactions (Rigaud, Perrot‐Minnot, & Brown, 2010). This is partly because studies concerning multiple parasite species interactions are much harder to perform and interpret. Indeed, interactions between parasitic species within host organisms can take very different forms even in the simplest case when a host harboring one parasite is subsequently infected with another parasite (see Cox, 2001; Thomas, Adamo, & Moore, 2005; Cézilly, Perrot‐Minnot, & Rigaud, 2014; Vaumourin, Vourc'h, Gasqui, & Vayssier‐Taussat, 2015 for review and theoretical considerations).

Thus, the presence of certain parasitic species in a host can be more or less beneficial or deleterious to other parasites that subsequently attempt to infect that same host depending on the transmission ecology of the co‐occurring parasite species and host‐mediated effects (Cox, 2001; Thomas et al., 2005; Vaumourin et al., 2015). A parasite which has already entered a host organism, for example, can influence (directly or indirectly) the success of subsequent infections by other parasitic species (see Cox, 2001; Johnson, de Roode, & Fenton, 2015; Kotob, Menanteau‐Ledouble, Kumar, Abdelzaher, & El‐Matboulim, 2016; Pedersen & Fenton, 2007; Vaumourin et al., 2015). This influence may lead to a positive interaction, i.e., the presence of one parasite may facilitate subsequent infections by other parasites, or negative interaction between parasitic species (Hoverman, Hoye, & Johnson, 2013; Jackson, Pleass, Cable, Bradley, & Tinsley, 2006; Klemme & Karvonen, 2017; Vaumourin et al., 2015).

In general, mechanical damage and immunity are likely to be among the most important factors influencing the success of the infection in preinfected hosts (Pedersen & Fenton, 2007). Thus, in humans preinfection with herpes simplex virus type 2 causes damage to mucous membranes paving the way to HIV infection (Vaumourin et al., 2015 and references therein). In fish, similar mechanical lesions caused by the ectoparasitic crustacean *Argulus coregoni* increase host susceptibility to the pathogenic bacterium *Flavobacterium columnare* (Bandilla, Valtonen, Suomalainen, Aphalo, & Hakalahti, 2006). By contrast, the infection with one myxozoan species can prevent the subsequent invasion of other myxozoan species presumably due to cross‐immunity (Kotob et al., 2016). Likewise, preinfection also can have no impact on subsequent infection as demonstrated by Karvonen, Seppälä, and Valtonen (2009) in their study of two species of trematodes from the genus *Diplostomum* infecting rainbow trout and by Chowdhury et al. (2018) in a freshwater pearl mussel study. Although the impact of preinfection by one parasite on the infection success of other parasites has been studied in fish (Kotob et al., 2016), these studies, mainly concerned interactions between microparasites. Preinfection studies with macroparasites have received far less attention. Kotob et al. (2016), for example, reviewed data on parasitic interactions in fish, but did not present any examples of macroparasite–macroparasite interactions (but see Karvonen et al., 2009).

Parasitic associations, i.e., positive or negative correlations between infection intensities, are rather commonly reported from wild hosts (Booth, 2006; Johnson & Buller, 2011; Karvonen et al., 2009; Pedersen & Fenton, 2007; Rigaud et al., 2010). In natural conditions, however, true interparasitic interactions are often masked by positively or negatively correlated coinfection (Johnson & Buller, 2011). Consequently, associations between two species can disappear or change from positive to negative, when a controlled experiment is performed (e.g., Johnson & Buller, 2011; Karvonen et al., 2009). However, it is interactions, rather than associations, among parasites that play an important role in structuring populations and communities of both hosts and parasites (Rigaud et al., 2010; Vaumourin et al., 2015). In addition, the order in which different parasitic species or genotypes attack and enter the host can influence the interaction between parasites within the host individual (Hoverman et al., 2013; Klemme & Karvonen, 2017; Read & Taylor, 2001; Telfer et al., 2010). Therefore, an experimental approach is needed to reveal, whether there is real interaction between parasites or, at least, evaluate the consequences of such interactions.

The freshwater pearl mussel, *Margaritifera margaritifera* is a freshwater bivalve critically endangered through its native range (Geist, 2010; Lopes‐Lima et al., 2017). This bivalve has an obligate parasitic larval stage, the glochidium, which attaches to gills of salmonid fishes, such as Atlantic salmon (*Salmo salar*) and brown trout (*Salmo trutta*) (Salonen, Marjomäki, & Taskinen, 2016; Salonen et al., 2017; Taeubert & Geist, 2017). The latter fish is the exclusive host for freshwater pearl mussel glochidia in many Central European populations (Geist, Porkka, & Kuehn, 2006; Taeubert & Geist, 2013). Often freshwater pearl mussel glochidia are considered to have a positive or, at most, a very weak negative effect on the host's health (Ziuganov, Zotin, Nezlin, & Tretiakov, 1994; Ziuganov, 2005; but see Taeubert & Geist, 2013). The latter is perhaps more likely to be true since freshwater pearl mussel glochidia demonstrate more than sevenfold growth in the course of 8–10‐month development on the fish gills with a substantial nutrient transfer from fish to glochidia and a shift in the stable isotope composition during the mussel's development (Denic, Taeubert, & Geist, 2015). In addition, *M. margaritifera* glochidia cause a pronounced immune response in their hosts, increase metabolic rates and hematocrit levels and hamper swimming and respiratory performance in brown trout (Chowdhury, Salonen, Marjomäki, & Taskinen, 2018; Filipsson et al., 2017; Österling, Ferm, & Piccilo, 2014; Taeubert & Geist, 2013; Thomas, Taylor, & Garcia de Leaniz, 2014). Impairment of the respiratory capacity of host fish is especially important since gills can be a place where other infectious agents enter the host. It has been demonstrated that exposure of fish to pathogens and parasites increases with the ventilation rate (Mikheev, Pasternak, Valtonen, & Taskinen, 2014). Therefore, freshwater pearl mussel glochidia are likely to be a suitable predecessor for parasitic species infecting brown trout through gills (i.e., increase their infection success). However, this assumption has never been tested experimentally. Moreover, to our knowledge, there were no previous studies, where the interaction between glochidia and other parasites was tested experimentally.

The eye fluke trematode *Diplostomum pseudospathaceum* is a ubiquitous species (Klemme & Karvonen, 2017) which is a common parasite of a wide array of fishes, such as many cyprinid and salmonid fishes including brown trout in natural environments (Betterton, 1974; Rolbiecki, Sciazko, & Schütz, 2009; Valtonen & Gibson, 1997). *D. pseudospathaceum* has three hosts in its life cycle (Seppälä, Karvonen, & Valtonen, 2004). Freshwater snails (often *Lymnaea stagnalis*) are the first intermediate hosts, freshwater fishes are the second intermediate hosts and fish‐eating birds are the definitive hosts. Infected snails produce thousands of cercariae (the trematode's dispersal stage) which penetrate fish epithelium primarily through gills (Mikheev et al., 2014). Therefore, the fluke's infection success strongly depends on the amount of water pumped through fish gill chambers (Mikheev et al., 2014). After penetrating fish epithelium, *D. pseudospathaceum* moves toward the eye, the place of its final development into the metacercarial stage within the eye lens.

In contrast to *M. margaritifera*,* D. pseudospathaceum* is likely to be an unsuitable “predecessor” for other parasitic species. In the eye lens, flukes are unprocurable for the host immune system (Höglund & Thuvander, 1990; Wegner, Kalbe, & Reusch, 2007). Therefore, there is no need for them to suppress the host's immune system to avoid its negative influence on the parasite. Although such immunity regulation is known for numerous other parasitic species (Cox, 2001; Maizels & McSorley, 2016; McSorley, Hewitson, & Maizels, 2013), it is unlikely to be the case for *D. pseudospathaceum*. Moreover, Höglund and Thuvander (1990) suggested that *D. pseudospathaceum* nonspecifically enhances host immunity. Although specific antibody production was not recorded, they found that preinfection with *D. pseudospathaceum* induces some degree of protective immunity against subsequent infections with these parasites, suggesting some kind of nonspecific immunity enhancement caused by eye flukes (Höglund & Thuvander, 1990; see also Karvonen, Paukku, Seppälä, & Valtonen, 2005).

In our study, we sequentially infected brown trout with glochidia of the freshwater pearl mussel and cercariae of the eye fluke and vice versa. We hypothesized that fish preinfected with freshwater pearl mussel glochidia are more vulnerable to the cercarial infection by *D. pseudospathaceum* because glochidia can impair respiratory gas exchange and elevate resting respiratory rate of infected fish (Thomas et al., 2014), thereby increasing the amount of water (and cercariae) pumped through the gill chamber. To test this hypothesis we repeated the experiments twice using different (high and low) doses of cercariae during the infection. We also hypothesized that brown trout preinfected with *D. pseudospathaceum* would be less or similarly vulnerable to the subsequent freshwater pearl mussel glochidia infection because *D. pseudospathaceum* does not change or even enhances the performance of the host immune system. In this experiment we also manipulated the timing of preinfection with trematode (i.e., 20 hr vs. 2 weeks before the subsequent infection by glochidia). This took into account the possible host mediated effects because since an immune response may occur in fish soon after the infection *D. pseudospathaceum* is likely to cause in fish, whereas once it has migrated to the eye lens, it is “invisible” to the host's immune system (Höglund & Thuvander, 1990; Wegner et al., 2007).

## 2 MATERIAL AND METHODS

### 2.1 Experimental infection procedures

#### 2.1.1 Fish preinfected with freshwater pearl mussel glochidia

Young‐of‐the‐year brown trout (*Salmo trutta*) from the Iijoki (Finland) sea‐run and Rautalampi (Finland) lake‐run stock were collected from the Natural Resources Institute Finland (Luke) in Taivalkoski and the Luke in Laukaa (Finland), respectively, in the end of August 2014 and transported to the Konnevesi Research Station of the University of Jyväskylä, Finland. Fish from each stock were placed into four (altogether eight) 163‐L flow‐through tanks. Fish were acclimated in the laboratory for 3 weeks and then some were mass‐infected with freshwater pearl mussel glochidia (exposure dose = 5,000 glochidia per fish) that originated from the Livojoki river (Table 1a). The procedure of the glochidia collection and exposure to fish was similar to Chowdhury et al. (2018). To collect glochidia we placed adult freshwater pearl mussels in plastic buckets filled with 5 L of river water for 30 min on the day of infection. The mussels were returned to the river after incubation and the glochidial suspension was transported to the Konnevesi research station.

**Table 1:** Experimental design. (a) Preinfection with *M. margaritifera* glochidia and (b) Preinfection with *D. pseudospathaceum*. Fish numbers (*n*) obtained after the exclusion of individuals with unclear infection state (see the text)

Preinfection with glochidia	Treatment	Exposure to cercariae	Fish mass, g, mean ± *SD*
(a) *M. margaritifera* → *D. pseudospathaceum* experiment			
Exposure dose = 5,000 glochidia/fish	Infected (*n* = 36)	High dose (300 cercariae/fish)	7.03 ± 2.17
	Control (*n* = 29)		
	Infected (*n* = 28)	Low dose (200 cercariae/fish)	20.10 ± 4.66
	Control (*n* = 55)		
	September, 2014	July, 2015	

Preinfection with cercariae	Treatment	Exposure to glochidia	Fish mass, g, mean ± *SD*
(b) *D. pseudospathaceum* → *M. margaritifera* experiment			
Exposure dose = 250 cercariae/fish	Preinfected 20 hr before glochidial infection (*n* = 15)	Exposure dose = 1,500 glochidia/fish	21.58 ± 4.65
	Preinfected 2 weeks before glochidial infection (*n* = 48)		
	Control (*n* = 56)		
	August, 2015		

Brown trout were mass‐exposed to glochidia in maintenance tanks, where the water volume was reduced to 70 L and water‐flow turned‐off. A similar procedure (i.e. mass‐exposure in 70 L of still water) was used in all experimental infections mentioned throughout the paper. Though individual exposure is sometimes recommended (Douda, 2013 and references therein), simultaneous exposure of the group of fish is commonly used in experimental parasitological practice (e.g., Gopko, Mikheev, & Taskinen, 2015, 2017; Seppälä, Karvonen, & Valtonen, 2008; Seppälä et al., 2004; Taeubert, Gum, & Geist, 2013; Taeubert, Martinez, Gum, & Geist, 2012). Such “mass‐exposure” approach is especially logical when studying fish, which spend a substantial amount of time in shoals (Taeubert et al., 2013) as juvenile salmonids do (Brännäs, Jonsson, & Lundqvist, 2003; Hicks & Watson, 1985). The exposure time was 1 hr, average exposure dose ~5,000 glochidia per fish, and water temperature was 14.2°C. Control animals were treated identically to preinfected fish with the exception that instead of cercariae they were exposed only to water. These methods were used during all infection procedures.

After exposure, brown trout were marked using fin clipping and randomly reallocated according to their original stock so that all eight tanks received a similar amount of infected and control fish. In four tanks the right fin was notched in control fish, while the left fin was notched in fish exposed to glochidia. In four other tanks, opposite fins were notched in control and infected fish. Though notching can cause an increased immune response in fish, this effect is likely to be relatively short term. Henrich, Hafer, and Mobley (2014) found out that the effect of spine clipping on host immunity became indistinguishable 2 weeks after the procedure. In our study, we marked fish 3 weeks after the exposure to glochidia (i.e., 9 months before the exposure to *D. pseudospathaceum* cercariae). Therefore, it is unlikely that fin notching could have had a substantial effect on the success of trematode infection. In addition, fish were fin‐notched both in infected and control groups.

Following Taeubert et al. (2012, 2013) five fish from each tank were sacrificed randomly 3 days postexposure to check for infection success. We found successful infection (ranging from 19 to 782 glochidia per fish with mean ± *SD* = 313.0 ± 239.3) in glochidia exposed fish, while fish from the control group had no glochidia. Fish were subsequently maintained for 10 months in four similar 163‐L round flow‐through tanks. Throughout the experiment control fish were treated identically to infected fish.

In June–July 2015 brown trout from two randomly chosen tanks with fish from each stock were exposed to *D. pseudospathaceum*. The fish were exposed in summer to make experimental conditions synchronous with their natural seasonal occurrence. In Finland detachment of *M. margaritifera* glochidia from fish gills happens in June–July (Salonen & Taskinen, 2017). Therefore, damage caused to fish gills by glochidial excystment and the increase in host vulnerability to subsequent infections is likely to be most pronounced in this period of the year. In addition, snails infected with *D. pseudospathaceum* are starting to produce cercariae actively when water temperatures are above 10°C (Lyholt & Buchmann, 1996; Voutilainen, Taskinen, & Huuskonen, 2010). Therefore, in boreal ecosystems of the northern hemisphere the probability of interactions between *M. margaritifera* glochidia and *D. pseudospathaceum* eye flukes within hosts is especially high in summer.

The exposure procedure was generally similar to Gopko et al. (2015). In brief, brown trout were exposed to the mixture of cercariae shed by 10 freshwater snails *Lymnaea stagnalis*. In our study, we did not identify parasites using molecular methods. However, previous studies showed that *L. stagnalis* snails are typically infected with *D. pseudospathaceum* (e.g. Rellstab, Louhi, Karvonen, & Jokela, 2011; Selbach, Soldánová, Georgieva, Kostadinova, & Sures, 2015). For instance, in Finland (including Lake Konnevesi) *L. stagnalis* is infected with *D. pseudospathaceum* (Louhi, Karvonen, Rellstab, & Jokela, 2010; Rellstab et al., 2011).

Cercariae used in our experiments were no older than 5 hr. The exposure time was 30 min which is similar to previous studies (e.g., Gopko et al., 2015, 2017; Klemme & Karvonen, 2016, 2017; Mikheev, Pasternak, Taskinen, & Valtonen, 2010; Mikheev et al., 2014; Seppälä et al., 2004). During the exposure water levels in each tank were decreased to 70 L and water flow was turned off. After the exposure, water flow was turned on and water volumes in the tanks were again increased to 163 L.

We repeated the experiment twice with a two‐week interval. In first two (out of four) randomly chosen tanks, fish from Iijoki stock were exposed to the low infection dose (200 cercariae per fish) at 12.5°C. Such a dose, however, resulted in lower metacercariae numbers in fish eye lenses than we planned (some fish were uninfected) (see Section 2.1). Therefore, we repeated the experiment with a higher doses of cercariae during the exposure (300 cercariae per fish, 12.8°C). In this high dose experiment we used fish from the Rautalampi stock.

After the *D. pseudospathaceum* infection (on 7th and 12th days in low and high dose infection experiment, respectively), fish were killed with an overdose of 0.01% MS 222 (Sigma Chemical Co., St Louis, U.S.A.), weighed and dissected. It is improbable that a small difference in the periods between infections and dissections influenced the results of dissections. This is because the *Diplostomum* metacercarial mortality rate within host lenses is likely to be extremely low, while the life span is very long, with a maximum of 4 years (Klemme & Karvonen, 2017; Shigin, 1980). The number of *D. pseudospathaceum* metacercariae and freshwater pearl mussel glochidia were counted using a dissection microscope (32× magnification).

#### 2.1.2 Fish preinfected with *D. pseudospathaceum*

A reciprocal experiment (brown trout preinfected with eye flukes and then infected with glochidia at 14.2°C) was conducted in August 2015. One hundred and twenty young‐of‐the‐year brown trout, “Rautalampi” stock, were obtained from the Natural Resources Research Institute (Luke) fish farm at Laukaa (Table 1b). Exposure procedures were similar to the described above. The exposure dose was 250 cercariae per fish. Fish were divided into three groups: (a) exposed to cercariae 2 weeks prior to the glochidia infection, (b) exposed 20 hr prior to the glochidia infection and (c) control fish exposed only to lake water). The 20 hr group was added because eye flukes usually need 24–48 hr to migrate to the host eye lens. Within‐host migration is the only period when the parasite is vulnerable to the fish's immune system (Höglund & Thuvander, 1990). Then all fish were infected with the glochidia of the freshwater pearl mussel. The exposure dose of glochidia infection was 1,500 glochidia per fish and an exposure time of 1.5 hr. Glochidia were collected from Jukuanoja, a tributary of the Iijoki River and were transferred to Konnevesi research station on 31 of August 2015. Fish were dissected on the 13th day after the infection with trematodes. Metacercariae and glochidia were counted using a dissection microscope (32× magnification).

Throughout the experiments, fish were daily fed with commercial food pellets (1.5 mm size, Nutra Parr LB, Norway). The experiments were conducted with the permission of the Centre for Economic Development, Transport and Environment of South Finland (license number ESAVI/6759/04.10.03/2011).

### 2.2 Statistical analysis

During dissections we found that not all fish marked as preinfected with freshwater pearl mussel really had glochidia on their gills. It could be a result of unsuccessful infection or active dropping off the host gill by the mature parasites. The latter is more likely because the freshwater pearl mussel glochidia prevalence in experimental infections usually is close to 100% (e.g., Taeubert & Geist, 2013). By contrast, the duration of glochidial development on the fish gills is highly variable (from 3 weeks to 10 months) and depends on several factors including parasites’ compatibility with the host (Geist et al., 2006; Marwaha, Jensen, Jakobsen, & Geist, 2017). Since we could not estimate how much time passed since glochidia dropped off the gills, those fish that were marked as infected, but did not have any glochidia on their gills were considered as being of unknown status and were excluded from the subsequent analysis (23 and 44 fish in low and high dose trematode infection treatment, respectively). However, when these fish are included in the analyses, statistical effects remain significant.

R software was used for all statistical analyses (R Core Team, 2017). Plots were drawn using the “ggplot2” package (Wickham, 2009).

In fish preinfected with glochidia, we started with a general linear model (GLM) where the Box–Cox transformed *D. pseudospathaceum* infection intensity was a response variable, infection state (preinfected with glochidia or not) and experiment (low vs. high exposure dose) were factors and fish mass was a covariate. Response variable transformation was needed since the residuals’ distribution of our model strongly violated the normality assumption (Shapiro–Wilk (S–W) test, *W* = 0.928, *p* < 0.001). After the transformation, data distribution became close to normal (S–W test, *W* = 0.984, *p* = 0.09). Since experiments with low and high exposure doses were conducted with a time lag and can be considered independent, we also fitted separate models for each experiment. In the high dose treatment, we started with a general linear model (GLM) where the *D. pseudospathaceum* infection intensity was the response variable, infection state was a factor and fish mass as a covariate. However, mass did not have a significant influence on the eye fluke infection intensity and was excluded from the final model. A residual values distribution did not differ from normal (S–W test, *W* = 0.971, *p* = 0.13).

In the low dose treatment, the Shapiro–Wilk test showed that even after transformation there was a violation of the normality assumption in our data due to several obvious outliers seen on the Q–Q plot (S–W test, *W* = 0.944, *p* = 0.001). Therefore, we had to fit a robust regression based on M‐estimator with Huber's weights, with the tuning constant *k* = 1.345*σ* using MASS package in R programming software (Venables & Ripley, 2002). The f.robtest function in the sfsmisc package was used to compute robust *F*‐test and get *p*‐values (Maechler, 2017). The square root transformed intensity of the *D. pseudospathaceum* infection was a response variable. The Fisher's exact test was used to estimate whether the probability of getting infected in preinfected fish was higher than in the control group.

In the reciprocal experiment (where fish were preinfected with *D. pseudospathaceum*), we used a generalized linear model with a log link function and Gaussian error structure, where the glochidial infection intensity was the dependent variable, infection state was a factor and fish mass was a continuous predictor. Post hoc comparisons were done using a *glht* function in the “multcomp” package (Hothorn, Bretz, & Westfall, 2008). *p*‐Values were adjusted using Bonferroni corrections.

## 3 RESULTS

### 3.1 Fish preinfected with freshwater pearl mussel glochidia

**Figure 1:**
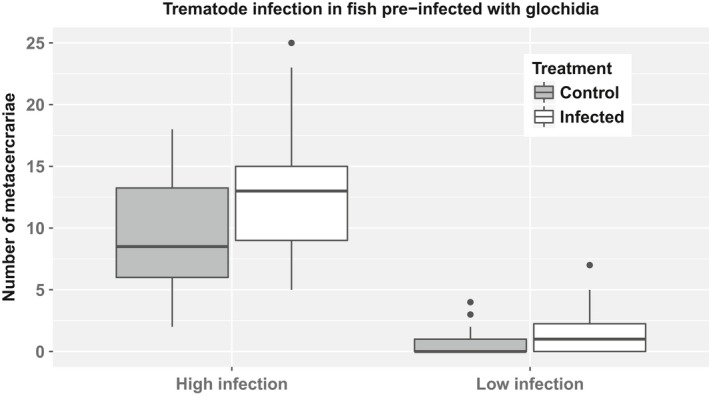
Both in the low and the high cercaria dose treatment, brown trout preinfected with freshwater pearl mussel glochidia were more vulnerable to infestation by *D. pseudospathaceum* trematode cercariae than control fish. The “box” represents the interquartile range (IQR) of the *D. pseudospathaceum* metacercaria infection intensities within groups with median (black line). Whiskers extend from the highest to lowest values within 1.5*IQR. Suspected outliers, i.e., all observations lying outside 1.5*IQR, are shown as dots

There was a significant effect of the preinfection with glochidia on the intensity of the subsequent eye‐fluke infection (Table 2; Figure 1). As hypothesized, fish preinfected with glochidia were more vulnerable to the subsequent infection with trematodes than control fish (Figure 1). The effect of the covariate fish mass was statistically significant (Table 2), indicating that the intensity of the trematode infection decreased with host size. Finally, as hypothesized, higher exposure dose of cercariae resulted in higher infection intensities in brown trout (Table 2, Figure 1). The effects of interactions were nonsignificant and therefore interactions were excluded from the final model.

**Table 2:** The GLM demonstrated that there were significant effects of treatment (preinfection with glochidia), fish mass and exposure dose on the success of the subsequent *D. pseudospathaceum* infection (Box–Cox transformed values). Preinfected fish were more vulnerable to the infection compared with controls. In addition, bigger brown trout had lower eye fluke infection intensity than smaller ones. As expected lower exposure dose of trematode's cercariae result in lower infection intensities in brown trout

Source	Estimate	*SE*	*t*‐Value	*p*‐Value
Treatment (infected)	0.323	0.082	3.913	0.0001
Mass	−0.031	0.011	−2.874	0.0047
Experiment (low exposure dose)	−1.586	0.160	−9.944	<0.0001

If low and high dose treatments were analyzed separately, the effect of the glochidial preinfection on the success on subsequent trematode infection was still significant. In the high dose treatment (fish infected with a high dose of cercariae) all brown trout (*N* = 64, fish mass mean ± *SD* = 7.03 ± 2.17 g) were successfully infected with *D. pseudospathaceum* metacercariae (range 2–25). In the high dose treatment, the mean intensity of *D. pseudospathaceum* infection in fish preinfected with freshwater pearl mussel glochidia was significantly higher compared with the control group (GLM ANOVA, *t* = 2.7, *p* = 0.008; Figure 1). When included in the model, fish mass did not significantly influence the *D. pseudospathaceum* infection intensity (*p* > 0.6). Weights of control and preinfected brown trout were similar (GLM ANOVA, *t* = 0.03; *p* = 0.98).

In the low dose treatment (fish infected with a low dose of cercariae) only 28 brown trout out of 83 (*N* = 83, fish mass mean ± *SD* = 20.10 ± 4.66 g) were infected with the eye fluke. In the low dose treatment, the probability of being infected was significantly higher in preinfected fish compared with the control group (Fisher's exact test, *p* = 0.016).

The robust regression based on M‐estimation showed that in the low dose experiment both preinfection with glochidia and fish mass significantly influence the *D. pseudospathaceum* infection intensity. Fish preinfected with *M. margaritifera* glochidia were significantly more vulnerable to the subsequent infection with *D. pseudospathaceum* trematode compared with control fish (Robust *F* = 7.113, *p* = 0.009). The infection intensity of *D. pseudospathaceum* decreased with the fish size in the low dose experiment (Robust *F* = 10.47, *p* = 0.002). Fish mass (mean ± *SD* = 21.58 ± 4.65 g) was similar in control and preinfected fish (GLM ANOVA, *t* = 1.25; *p* = 0.22).

Surprisingly, in both treatments there was no significant relationship between the intensity of glochidia preinfection and the intensity of *D. pseudospathaceum* subsequent infection, when only preinfected fish were taken into consideration (Pearson's correlation coefficient, *r* = −0.13, *p* = 0.53, *N* = 28 and *r* = 0.30, *p* = 0.12, *N* = 28, high and low dose treatment, respectively). There was also no significant correlation between the fish mass and the glochidia infection intensity (*r* = 0.19, *p* > 0.33, *N* = 28 in the high dose treatment and *r* = 0.15, *p* = 0.47, *N* = 28 in the low dose treatment). When the two datasets (glochidia‐infected fish from high and low dose treatment) were merged and possible confounding variables (fish mass and treatment) were taken into account, the result was similar (see Supporting Information Table S1). Intensities of the glochidial infection were (mean ± *SD*) 376.5 ± 275.3 and 438.7 ± 286.7 glochidia/fish in low and high dose treatment respectively.

### 3.2 Fish preinfected with *D. pseudospathaceum*

**Figure 2:**
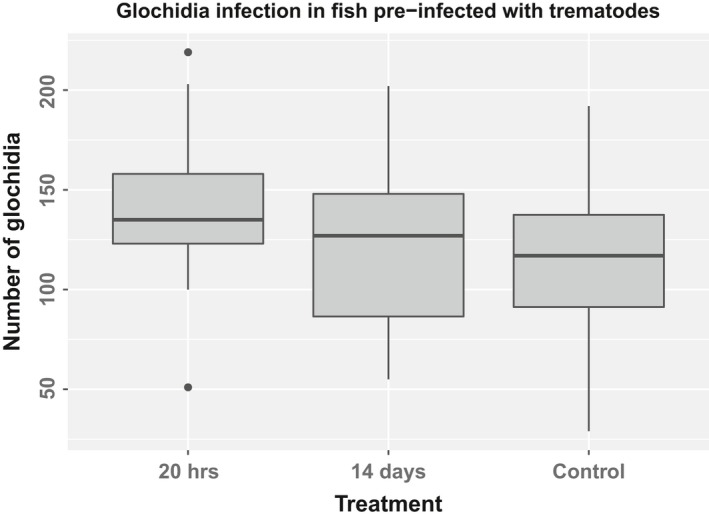
Fish preinfected with the *D. pseudospathaceum* were more vulnerable to the freshwater pearl mussel glochidia infection compared with control fish, when the preinfection took place 20 hr before the exposure to glochidia. Two weeks after preinfection, however, this pattern did not occur. For an explanation of the box, vertical line and whiskers, see Figure 1. Suspected outliers, i.e., all observations lying outside 1.5*IQR, are shown as dots. These points were included in the analysis, but when a robust regression was used, results of statistical tests remained similar (see the Supporting Information Table S2)

The GLM followed by post hoc comparisons showed that fish preinfected with *D. pseudospathaceum* 20 hr before the subsequent infection with *M. margaritifera* glochidia were significantly more vulnerable to the glochidial infection compared with control fish (Figure 2, Table 3). On the other hand, fish preinfected with *D. pseudospathaceum* cercariae14 days prior to the infection with glochidia had glochidial loads that did not differ significantly from those of the control fish. Glochidial loads significantly increase with the increase of the fish weight (Table 3). Fish weights did not differ between the treatments (ANOVA *F*_2,116_ = 0.097; *p* = 0.91). The intensities of glochidial infection were moderate (mean ± *SD* glochidia/fish was 138.9 ± 41.5 in 20 hr earlier preinfected, 119.4 ± 36.8 in 14 days earlier preinfected and 114.6 ± 38.9 in control fish respectively, Figure 2, see also Supporting Information Figure S1).

**Table 3:** Results of the GLM and subsequent post hoc comparisons examining the effect of the preinfection with *D. pseudospathaceum* on brown trout vulnerability to subsequent freshwater pearl mussel glochidia infection in brown trout

Source	Estimate	*SE*	*t*‐Value	*p*‐Value
Results of generalized linear model				
Treatment 1 day	0.202	0.075	2.69	0.008
Treatment 14 days	0.030	0.058	0.52	0.602
Mass	0.116	0.023	5.10	<0.0001

Comparison	Estimate	*SE*	*z*‐Value	*p*‐Value
Multiple comparisons				
20 hr–14 days	−0.172	0.076	−2.25	0.073
Control–14 days	−0.030	0.058	−0.52	1.000
Control–20 hr	0.202	−0.075	−2.69	0.022

## 4 DISCUSSION

The results of our study indicate that the infection with glochidia of the freshwater pearl mussel can predispose fish to a concomitant infection with other parasitic species. Brown trout preinfected with freshwater pearl mussel glochidia were infected with more *D. pseudospathaceum* during the subsequent experimental infection compared with control fish. We repeated the experiment with two different doses of cercariae during the subsequent infection and both times the result was similar. In addition, in the low dose treatment—i.e., when a low dose of cercariae was used in the subsequent infection—fish preinfected with glochidia were more likely to become infected with *D. pseudospathaceum* than the control fish. The numbers of freshwater pearl mussel glochidia in preinfected fish (~400 glochidia per fish on average) were within the range of glochidia numbers found in natural salmonid populations (e.g., Salonen & Taskinen, 2017; Ziuganov, 2005), thereby providing a close biological relevance to the experimental findings.

Previous studies have demonstrated that freshwater pearl mussel glochidia constitute a respiratory burden for the infected fish (Thomas et al., 2014). In turn, *D. pseudospathaceum* cercariae enter their hosts mainly through gills and increased ventilation rate also increases infection rate of *D. pseudospathaceum* infection in fish (Mikheev et al., 2014). Therefore, an enhanced ventilation rate in fish infected with glochidia is likely to be a plausible mechanism explaining their higher vulnerability to the *D. pseudospathaceum* infection. In addition, June–July, when subsequent infections with *D. pseudospathaceum* cercariae were performed, is the period when freshwater pearl mussel glochidia detach from fish (Salonen et al., 2017)—an event that typically damages gills as glochidial cysts rupture. This should further increase the vulnerability of fish to *D. pseudospathaceum* cercariae.

It has been proposed earlier that the relationship between *M. margaritifera* glochidia and fish host is neutral or mutualistic (e.g., Ziuganov, 2005; Ziuganov et al., 1994), but our results provide evidence that the relationship at least moderately antagonistic. Therefore, our data is consistent with previous studies, which demonstrated that brown trout infected with freshwater pearl mussel glochidia perform generally worse compared to uninfected fish (Filipsson et al., 2017; Österling et al., 2014; Taeubert & Geist, 2013; Thomas et al., 2014). This is in line with the fact that freshwater pearl mussel glochidia grow intensively on the fish gills and obtain nutrients from their hosts (Denic et al., 2015). However, Ziuganov (2005) found lower prevalence of saprolegniosis in Atlantic salmon infected by freshwater pearl mussel glochidia than in noninfected salmon and suggested an increase in nonspecific resistance in fish host caused by freshwater pearl mussel glochidia infection. Our results do not rule out this possibility, but indicate that either the proposed immunity enhancement does not include macroparasites, such as trematode cercariae, or that negative effects of gill damage due to detaching glochidia override a possible positive effect of the immune enhancement. Therefore, it would be interesting to perform subsequent infections with fungal, bacterial and viral pathogens in addition to parasites and at different times after the preinfection with freshwater pearl mussel glochidia. Shortly after the preinfection and at the time of glochidia detachment, the negative effect of gill damage should prevail, but in the course of glochidia development on gills or after the detachment period the possible immunity enhancement should be observed.

A concomitant infection by trematodes caused by preinfection with freshwater pearl mussel glochidia may hamper the host directly, e.g., by utilizing additional host resources and/or loading its immune system. However, indirect effects of such concomitant infection can be even more severe. *Diplostomum* eye flukes, for example, can cause cataracts in fish eye lenses hampering fish vision and their ability to forage (Karvonen, Seppälä, & Valtonen, 2004; Owen, Barber, & Hart, 1993). More importantly, *D. pseudospathaceum* along with many other trophically transmitted parasite species (see Poulin, 2010 for the review) are able to manipulate host behavior predisposing fish to predation by the definitive host—fish‐eating bird (e.g., Crowden & Broom, 1980; Gopko et al., 2015, 2017; Mikheev et al., 2010; Seppälä et al., 2004, 2008). Therefore, by increasing the probability of concomitant infection freshwater pearl mussel glochidia can hamper their hosts in more subtle ways than it was suggested earlier (Filipsson et al., 2017; Österling et al., 2014; Taeubert & Geist, 2013; Thomas et al., 2014) which are not easy to detect without conducting comprehensive experimentation.

Salmonids and freshwater pearl mussels inhabit running waters while the snail host of *D. pseudospathaceum*,* L. stagnalis*, prefers standing waters. Thus, co‐occurrence of freshwater pearl mussel glochidia and *D. pseudospathaceum* in a salmonid host is probably infrequent, even though the geographic the distribution of the two species largely overlaps. However, many river systems have lentic sections or a lake as the source of the river. Because trematode cercariae can aggregate as dense clouds in the surface layer (Horák et al., 2015) and potentially can be carried over long distances by the wind, the flow of *D. pseudospathaceum* cercariae into freshwater pearl mussel rivers is possible. Indeed, today many freshwater pearl mussel rivers have been dammed for hydroelectric power, or modified for other purposes creating standing water reservoirs, ponds or stagnant water microhabitats favoring occurrence of *L. stagnalis*. Diplostomatids are masterful dispersers owing to their bird definitive host (see Louhi et al., 2010). Consequently, diplostomatid parasites are commonly recorded in brackish water and freshwater systems harboring fish and snails worldwide (Blasco‐Costa & Locke, 2017; Louhi et al., 2010; Valtonen & Gibson, 1997). In addition, the nutrient input to rivers is also increasing, which should favor *L. stagnalis*. These anthropogenic changes, together with climate warming, which should also benefit snails, probably increase the probability of the freshwater pearl mussel glochidia—*Diplostomum* coinfection in the future.

When fish preinfected with freshwater pearl mussel glochidia were subsequently infected with a low dose of cercariae, the number of *D. pseudospathaceum* metacercariae declined with fish mass, whereas in the high dose treatment no significant relationship was found. A possible explanation could be related to fish size. In the low dose treatment fish were about three times bigger compared to the high dose treatment. Although in the place of their final localization (eye lens) *D. pseudospathaceum* metacercariae are unprocurable for the host immune system, they are attacked by innate immunity while moving to the lens through host tissues (Höglund & Thuvander, 1990; Karvonen et al., 2005; Wegner et al., 2007). The bigger the fish, the longer the distance for the parasite to migrate to the eye lens and the longer the period when the parasite is thus vulnerable to a host immune response. It is necessary to mention, however, that we are unaware about studies, where a correlation between the fish size and time taken by *D. pseudospathaceum* to reach eye lenses was demonstrated.

The difference in fish sizes between experiments may be explained by different origin of brown trout used in the high‐ and low‐infection treatment. Since fish originated from different stocks, their tolerance to laboratory conditions (e.g., feeding regime, temperature, salinity etc.) can be slightly different, which in turn can lead to unequal growth rates during 10‐month maintenance in the laboratory. This also refers to the different *D. pseudospathaceum* infection intensities in fish from low‐ and high‐exposure experiments. Fish from different stocks (populations) may differ in their susceptibility to parasites (Bryan‐Walker, Leung, & Poulin, 2007; Hasu, Benesh, & Valtonen, 2009; Scharsack & Kalbe, 2014). However such difference is often a result of the evolution in different environmental condition, e.g., in lake (high parasitic pressure) compared to rivers (low parasitic pressure) (Scharsack & Kalbe, 2014), while in our study we were dealing with two river populations. Therefore, difference in parasitic load in two experiments is likely to a result of different exposure doses. However interpopulation differences also may play some role.

The intensity of the glochidial infection also did not correlate with the fish size both in the high and the low dose treatment, which is in agreement with previous studies (e.g., Thomas et al., 2014). Though in early stages (few weeks after the infection) bigger fish have more parasites, later this pattern disappears (Thomas et al., 2014). This is presumably because of uneven shedding of glochidia from different hosts due to differences in hosts’ immunity and/or nutrition and host–parasite compatibility (Marwaha et al., 2017).

A similar explanation can be applied to lack of the correlation between the glochidia and trematode infection intensity. Since glochidia are unevenly detached from gills (Marwaha et al., 2017) and damage to gill lamellae caused by glochidia (Thomas et al., 2014) may vary between fish due to the different immune response, pure glochidia numbers may not fully mirror the condition of the host respiratory system. Thus, increased host's vulnerability to the subsequent infection can be connected with damage caused by freshwater pearl mussel glochidia during the excystment. In such case *D. pseudospathaceum* infection intensity would correlate with the number of glochidia recently shed rather than with number of glochidia on gills. Another constraint, which can potentially obscure the relationship between glochidia and trematode infection intensities, is hosts’ individual differences in the innate immune response to the *Diplostomum* infection. Thus, Rauch, Kalbe, and Reusch (2006) found out that even within the same population fish can substantially differ in their vulnerability to *D. pseudospathaceum* and Kortet, Lautala, Kekäläinen, Taskinen, and Hirvonen (2017) observed between family differences in vulnerability to *D. pseudospathaceum* in fish.

Potentially, freshwater pearl mussel glochidia can also predispose their hosts to infectious agents other than eye flukes. For instance, a myxozoan endoparasite *Tetracapsuloides bryosalmonae* causes proliferative kidney disease (PKD) in salmonid fishes including brown trout (Vasemägi et al., 2017). PKD outbreaks have a strong economic and ecological importance (Okamura, 2016). Infection of the fish occurs via penetration of the gills by parasite spores (Vasemägi et al., 2017). Since at least in northern Europe freshwater pearl mussel and PKD are likely to be sympatric, increased respiratory burden connected with freshwater pearl mussel glochidia infection can also increase brown trout susceptibility to PKD, though, to our knowledge, this suggestion has never been tested explicitly.

Fish preinfected with *D. pseudospathaceum* differed in their vulnerability to the subsequent freshwater pearl mussel glochidia infection depending on when they were preinfected. Fish preinfected 2 weeks before the glochidia infection obtained a similar glochidia load to control fish, suggesting that the *Diplostomum* infection at least 2 weeks before exposure to freshwater pearl mussel glochidia does not increase host's vulnerability to glochidia. Generally, this result supports our initial hypothesis that *D. pseudospathaceum* metacercariae, which are unprocurable to the host's immune system within eye lenses, has no need to modulate or weaken the host's immune system as many other parasites do. By contrast, the intensity of freshwater pearl mussel glochidia infection in fish preinfected with *D. pseudospathaceum* just a 20 hr before the subsequent glochidia infection was higher than in control fish. During the first 24 hr after the infection eye flukes are moving through host tissues to eye lenses (Klemme & Karvonen, 2017) and therefore can cause an innate immune response (Wegner et al., 2007). According to the “optimal defense theory,” resource allocation to defense is flexible and there is a trade‐off between defense and other physiological functions related to the host (Stamp, 2003). For example, organisms, which are defending simultaneously from two threats (e.g., against the parasitic and predation threat), invest fewer resources towardsthe immune defense, when compared with organisms which are defending only against parasites (Rigby & Jokela, 2000). Similarly, when two unrelated parasites are entering the same host they can both benefit, because the host's immune system has to battle on two fronts simultaneously. Alternatively, it is possible that the penetration of *Diplostomum* cercariae could damage gill epithelium or change gill structure so that the subsequent infection by freshwater pearl mussel glochidia would be enhanced—but this effect lasts only for a short period. In theory, an exposure to *Diplostomum* cercariae shortly before the infestation by freshwater pearl mussel glochidia could be used in captive breeding programmes to facilitate infection success, for example if glochidia of this endangered mussel species are in short supply.

It is necessary to mention, that despite a clear positive ecological relationship between the two parasitic species demonstrated in our study, it is unlikely that both parasites can simultaneously increase their fitness due to this interaction. Since *Diplostomum* eye flukes and freshwater pearl mussels have very different life cycles, their notions about the future of the host's body are very different. For the eye fluke, a preferable scenario of the fish's future is predation by a fish‐eating bird, while the freshwater pearl mussel glochidia are interested in maintaining its host alive until glochidia will be metamorphosed into the juvenile stage and detached from gills. In spite of their positive relationship in sequential infections of fish, these parasites cannot be regarded as collaborators in the host's body and for each of them the facilitation of the subsequent infection success is probably no more than a byproduct of their own virulence. After establishing in the host, they can potentially start to hamper each other, for instance, by influencing host behavior in opposite directions (reviewed in Hafer, 2016). Therefore, more prolonged studies are needed to understand how the simultaneous infection with eye flukes and glochidia influences fitness of both parasitic species and their host.

## 5 CONCLUSIONS

In our study, we experimentally demonstrated how two common parasitic species of brown trout facilitate each other infection success by predisposing their host to subsequent infections. The infection with freshwater pearl mussel glochidia predisposes the fish host to the subsequent parasitic infection by the trematode entering the fish body trough gills during the respiration. Our findings demonstrate that our knowledge about the virulence of freshwater pearl mussel parasitic stages is still incomplete and that the negative influence of glochidia on host conditions is likely to be underestimated. In turn, the preinfection with *D. pseudospathaceum* can make fish more susceptible to the subsequent glochidial infection, but only, when the second parasite enters the host shortly after the first one. However the mechanism of this phenomenon remains unclear.

These data provide new evidence of how the timing and sequence of parasite exposure can influence infection success in a host–multiparasite system.

## CONFLICTS OF INTEREST

Authors declare that they have no conflicts of interest.

## AUTHOR CONTRIBUTIONS

All authors conceived the study. MC and MG conducted experiments. MC was mainly responsible for FPM part of the experiments, while MG for the trematode part. MG wrote the major part of the manuscript, while MC and JT added minor passages and edited the manuscript. JT supervised the study.

## DATA ACCESSIBILITY

Data are stored in the open figshare repository without any embargo and can be accessed following this link: https://doi.org/10.6084/m9.figshare.6063032.
