# Supplementary material for: Interactions between two parasites of brown trout (Salmo trutta): Consequences of preinfection
Source: Ecol Evol. 2018 Sep 29;8(20):9986–97. doi: 10.1002/ece3.4406 (PMC6206180; doi:10.1002/ece3.4406)
Supplement: Supplementary file 1 [file ECE3-8-9986-s001.docx]

**Table S1.** GLM with Gaussian error structure and identity link function did not reveal any significant relationship between Box-Cox transformed *Diplostomum pseudospathaceum* infection intensity (response variable), glochidia load (Glochidia) and fish mass (covariates). Experiment (high or low exposure dose of cercariae) was included in the model as a factor. Only fish pre-infected with glochidia were taken into account in this analysis.

| Source | Estimate | SE | t-value | p-value |
| --- | --- | --- | --- | --- |
| Glochidia | 0.0003 | 0.0003 | 1.078 | 0.286 |
| Mass | -0.0314 | 0.022 | -1.551 | 0.127 |
| Experiment (low dose) | -1.495 | 0.305 | -4.906 | < 0.0001 |


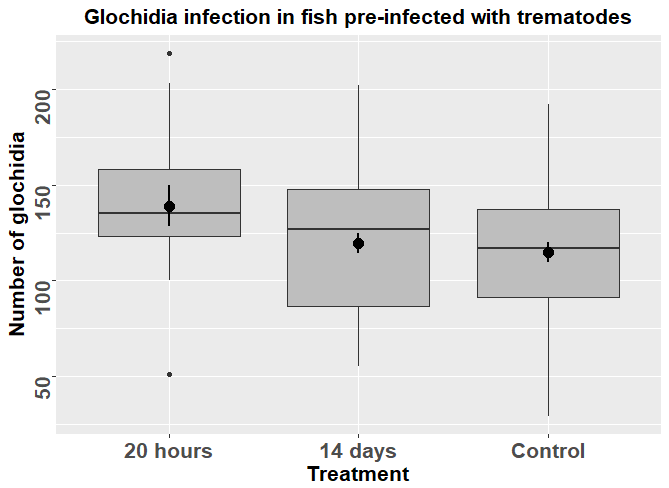


**Fig. S1**. Fish pre-infected with the *D. pseudospathaceum* were more vulnerable to the freshwater pearl mussel glochidia infection compared with control fish, when pre-infection took place 20 hours before the exposure to glochidia. However later (two weeks after the pre-infection) this pattern disappears. Means with +/- SE are presented as a large black dots with a thick vertical lines within boxes. The “*box*” represents the interquartile range (IQR) of the *D. pseudospathaceum* infection intensities within groups with median (black line). Whiskers extend from the highest to lowest values within 1.5*IQR. Suspected outliers, i.e. all observations lying outside 1.5*IQR, are shown as dots. These points were included in the analysis, however when a robust regression was used, results of statistical tests remain similar (see the Table s2).

**Table S2.** Robust regression based on M-estimator with Huber’s weights with the tuning constant k = 1.345σ showed that fish pre-infected with *D. pseudospathaceum* a 20 hours before the subsequent infection with glochidia were significantly more vulnerable to the glochidial infection compared with control fish. On the other hand, fish pre-infected with trematodes 14 days prior to the infection with glochidia had similar glochidial loads with control fish.

| Results of the robust regression | | | | |
| --- | --- | --- | --- | --- |
| Source | Estimate | SE | Robusr F | p-value |
| Treatment 20 hours | 26.92 | 10.60 | 6.48 | 0.012 |
| Treatment 14 days | 5.14 | 7.17 | 0.51 | 0.475 |
| Mass | 16.59 | 3.07 | 28.73 | <0.0001 |
